# Localizing Objective versus Subjective Cognition in Preclinical Alzheimer's Disease Using Atrophy Network Mapping

**DOI:** 10.1002/alz70856_099126

**Published:** 2025-12-24

**Authors:** Soyoung Lee, Sheena R Baratono, Grace T Burt, Nicole M Chiulli, Stephan T Palm, William J Drew, Benjamin S Zide, Michael D Fox, Reisa A. Sperling, Nancy J Donovan, Shan H Siddiqi

**Affiliations:** ^1^ Brigham and Women's Hospital, Boston, MA, USA

## Abstract

**Background:**

Alzheimer's disease (AD) pathologic changes, such as amyloid‐β (Aβ) accumulation, begin years before cognitive and functional impairment. However, it remains unclear if Aβ‐associated brain atrophy at this preclinical stage of AD maps to a distinct brain network associated with cognition. Identifying network‐level atrophy and its associations with cognition in preclinical AD is critical to understanding pathophysiology and could lead to more precise risk stratification.

**Method:**

Subjects included 1778 cognitively unimpaired older adults, aged 65‐85, from the Anti‐Amyloid in Asymptomatic Alzheimer's Disease (A4) and Longitudinal Evaluation of Amyloid Risk and Neurodegeneration (LEARN) studies. The data included objective cognitive function measured by Preclinical Alzheimer's Cognitive Composite (PACC), subjective cognitive decline measured by Cognitive Function Index reported by participants (CFI) as well as structural brain MRI and amyloid‐β (^18^F‐florbetapir) PET acquisitions. We generated a vertex‐wise general linear model of cortical thickness to create individualized atrophy maps for each participant with elevated Aβ, compared to participants with low Aβ as a control group. Then, we used a normative human connectome (*n* = 1000) to estimate the functional connectivity of each participant's unique atrophy pattern. We identified atrophy‐based brain networks associated with PACC and CFI scores, by performing linear regression between cognition and atrophy connectivity at each voxel. To test specificity, the resulting PACC map was adjusted for CFI, and the CFI map was adjusted for PACC.

**Result:**

Objective cognition (PACC) and subjective cognition (CFI) localized to brain networks. Objective cognitive impairment was more likely with atrophy patterns connected to the superior temporal gyrus (p_FWE_<0.05), and subjective cognitive impairment was more likely with atrophy patterns connected to the mid‐cingulate (p_FWE_<0.05). These findings remained significant adjusting for CFI and PACC respectively.

**Conclusion:**

We identified distinct brain networks associated with transitional objective and subjective cognition in preclinical AD using atrophy network mapping. These findings help elucidate early pathophysiology of AD and extend the use of this approach, previously applied to AD dementia, to localize transitional cognitive signs and symptoms in preclinical AD.

